# Supplementary material for: Annotation-preserving machine translation of English corpora to validate Dutch clinical concept extraction tools
Source: J Am Med Inform Assoc. 2024 Jun 27;31(8):1725–34. doi: 10.1093/jamia/ocae159 (PMC11258409; doi:10.1093/jamia/ocae159)
Supplement: ocae159_Supplementary_Data [file ocae159_supplementary_data.zip › ocae159_Supplementary_Data/Seinen2024_AnnotationPreservingCorpusTranslationAndValidation_Supplement_JAMIA_revised.docx]

Supplementary Material

Supplementary Tables

**Supplementary Table S1**. QuickUMLS settings in each MedSpaCy model.

| **Setting** | **Value** |
| --- | --- |
| overlapping_criteria | score (default) |
| threshold | 0.7 (default) |
| window | 10 |
| min_match_length | 3 (default) |
| similarity_name | Jaccard (default) |
| accepted_semtypes | List of UMLS semantic types that was used to annotate each corpus |

**Supplementary Table S2**. Details on the concept dictionaries for the Dutch and English MedSpaCy and unsupervised MedCAT models.

| **Statistic** | **MedSpaCy**  **English** | **MedSpaCy**  **Dutch** | **MedCAT: unsupervised**  **English** | **MedCAT:**  **Unsupervised**  **Dutch** |
| --- | --- | --- | --- | --- |
| Description | UMLS version 2022AB | UMLS  version 2022AB | *UMLS Small (A modelpack containing a subset of UMLS (disorders, symptoms, medications...). Trained on MIMIC-III)* | *UMLS Dutch v1.10 (a modelpack provided by UMC Utrecht containing UMLS entities with Dutch names trained on Dutch medical Wikipedia articles and a negation detection model repository/paper trained on EMC Dutch Clinical Corpus).* |
| Total no. of concepts | 4,612,422 | 4,074,856 | 573,284 | 383,628 |
| Total no. of terms | 9,803,663 | 8,354,007 | 1,950,576 | 1,123,689 |
| Total no. semantic types | 127 | 127 | 127 | 114 |

**Supplementary Table S3.** Five examples of sentences from the Mantra corpus with the English and Dutch references and the two (Google and GPT) Dutch machine translations (after annotation extraction), along with the translation metrics: BLEU and chrF. The agreement between the Google and GPT translations, measured using the same metrics, is also listed.

|  | **Text** | **BLEU** | **chrF** |
| --- | --- | --- | --- |
| English | *Always vary the site that you inject.* | - | - |
| Reference | *Wissel steeds van plaats die u voor de injectie gebruikt.* | - | - |
| Google | *Varieer altijd op de plaats waar u injecteert.* | 0 | 0.25 |
| GPT | *Wissel altijd de plaats af waar je injecteert.* | 0 | 0.32 |
| Google - GPT |  | 0 | 0.53 |
| English | *Reports of lupus and lupus-like syndromes, however, remain uncommon.* | - | - |
| Reference | *Gevallen van lupus of lupusachtige syndromen kwamen echter niet vaak voor.* | - | - |
| Google | *Rapporten van lupus en lupusachtige syndromen blijven echter zeldzaam.* | 0 | 0.56 |
| GPT | *Rapporten over lupus en lupusachtige syndromen blijven echter ongebruikelijk.* | 0 | 0.50 |
| Google - GPT |  | 0.56 | 0.76 |
| English | *Animal reproduction studies showed that degarelix caused infertility in male animals.* | - | - |
| Reference | *Uit voortplantingsonderzoek onder dieren is gebleken dat degarelix mannetjesdieren onvruchtbaar maakt.* | - | - |
| Google | *Dier reproductie studies toonden aan dat degarelix onvruchtbaarheid veroorzaakte bij mannelijk dieren.* | 0 | 0.42 |
| GPT | *Dier voortplanting studies toonden aan dat degarelix onvruchtbaarheid veroorzaakte bij mannelijke dieren.* | 0 | 0.52 |
| Google - GPT |  | 0.63 | 0.83 |
| English | *Patients weighing over 100 kg had a better response to the 90-mg dose.* |  |  |
| Reference | *Patiënten met een lichaamsgewicht van meer dan 100 kg vertoonden een betere respons op de dosis van 90 mg.* |  |  |
| Google | *Patiënten met een gewicht boven de 100 kg reageerden beter op de dosis van 90 mg.* | 0.39 | 0.51 |
| GPT | *Patiënten met een gewicht van meer dan 100 kg hadden een betere reactie op de dosis van 90 mg.* | 0.57 | 0.70 |
| Google - GPT |  | 0.44 | 0.69 |
| English | *When the results of all four studies were taken together, the average growth rate was 2.8 cm per year before treatment.* |  |  |
| Reference | *Wanneer de resultaten van de vier onderzoeken samen werden bekeken, lag de gemiddelde groei voor de behandeling bij 2,8 cm per jaar.* |  |  |
| Google | *Wanneer de resultaten van alle vier studies samen werden genomen, bedroeg de gemiddelde groei vóór behandeling 2,8 cm per jaar.* | 0.33 | 0.65 |
| GPT | *Wanneer de resultaten van alle vier de studies samen werden genomen, was de gemiddelde groeisnelheid 2,8 cm per jaar voor behandeling.* | 0.26 | 0.63 |
| Google - GPT |  | 0.50 | 0.80 |

**Supplementary Table S4.** Number of missing annotations in GPT's MM translations per semantic type, with the top-5 most frequently missed concepts.

CSV file: *TableS3_MM_GPT_missing_concepts_top5_per_semtype.csv*

**Supplementary Table S5.** Concept extraction performance per model type and corpus combination on the English version and on the (translated) Dutch versions of the three main corpora, measured by the three metrics: F1 score (F), precision (P), and recall (R).

|  |  | **MedMentions (MM)** | | | **Mantra (MT)** | | | **ShARe/CLEF (SC)** | | |
| --- | --- | --- | --- | --- | --- | --- | --- | --- | --- | --- |
| **Corpus language** | **Model type** | **P** | **R** | **F** | **P** | **R** | **F** | **P** | **R** | **F** |
| English | MedSpaCy | 0.40 | 0.79 | 0.53 | 0.55 | 0.59 | 0.57 | 0.50 | 0.81 | 0.62 |
|  | MedCAT unsupervised | 0.38 | 0.14 | 0.20 | 0.51 | 0.40 | 0.45 | 0.71 | 0.73 | 0.72 |
|  | MedCAT supervised | 0.30 | 0.83 | 0.44 | 0.42 | 0.91 | 0.58 | 0.28 | 0.85 | 0.42 |
| Dutch | MedSpaCy |  |  |  | 0.71 | 0.65 | 0.68 |  |  |  |
|  | MedCAT unsupervised |  |  |  | 0.64 | 0.49 | 0.55 |  |  |  |
|  | MedCAT supervised |  |  |  | 0.41 | 0.61 | 0.49 |  |  |  |
| Dutch (Google) | MedSpaCy | 0.38 | 0.79 | 0.51 | 0.67 | 0.70 | 0.69 | 0.50 | 0.74 | 0.60 |
|  | MedCAT unsupervised | 0.43 | 0.19 | 0.27 | 0.65 | 0.56 | 0.60 | 0.57 | 0.63 | 0.60 |
|  | MedCAT supervised | 0.31 | 0.47 | 0.37 | 0.41 | 0.73 | 0.52 | 0.37 | 0.73 | 0.49 |
| Dutch (GPT) | MedSpaCy | 0.33 | 0.73 | 0.45 | 0.67 | 0.66 | 0.66 | 0.53 | 0.77 | 0.63 |
|  | MedCAT unsupervised | 0.42 | 0.19 | 0.26 | 0.70 | 0.55 | 0.62 | 0.58 | 0.66 | 0.62 |
|  | MedCAT supervised | 0.29 | 0.41 | 0.34 | 0.39 | 0.67 | 0.49 | 0.39 | 0.75 | 0.51 |
